# Supplementary material for: Phagocytosis of Erythrocytes from Gaucher Patients Induces Phenotypic Modifications in Macrophages, Driving Them toward Gaucher Cells
Source: Int J Mol Sci. 2022 Jul 11;23(14):7640. doi: 10.3390/ijms23147640 (PMC9319206; doi:10.3390/ijms23147640)
Supplement: Supplementary file 1 [file ijms-23-07640-s001.zip › ijms-1774829-supplementary.pdf]

Phagocytosis of Erythrocytes from Gaucher Patients Induces Phenotypic Modifications in Macrophages, Driving Them toward Gaucher Cells

Lucie Dupuis [1], Margaux Chauvet [1], Emmanuelle Bourdelier [1], Michaël Dussiot [2], Nadia Belmatoug [3], Caroline Le Van Kim [1], Arnaud Chêne [1] and Mélanie Franco [1,*]

[1] INSERM, UMR_S1134, BIGR, Université Paris Cité and Université des Antilles, F-75015 Paris, France; lucie.dupuis@pasteur.fr (L.D.); margauxchauvet@hotmail.fr (M.C.); emmanuelle.bourdelier@inserm.fr (E.B.); caroline.le-van-kim@inserm.fr (C.L.V.K.); arnaud.chene@inserm.fr (A.C.)

[2] UMR_S1163, Sorbonne Paris Cité, Institut Imagine, Laboratoire d'Excellence GR-Ex, Université Paris Cité, F-75015 Paris, France; michael.dussiot@gmail.com

[3] APHP CRML Maladies Lysosomales, Service de Médecine Interne, Hôpital Beaujon, Sorbonne Université, F-92110 Clichy, France; nadia.belmatoug@aphp.fr

* *Correspondence:* melanie.franco@inserm.fr; Tel.: +33-(0)1-44-49-31-46

A.

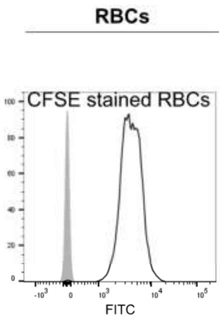

B.

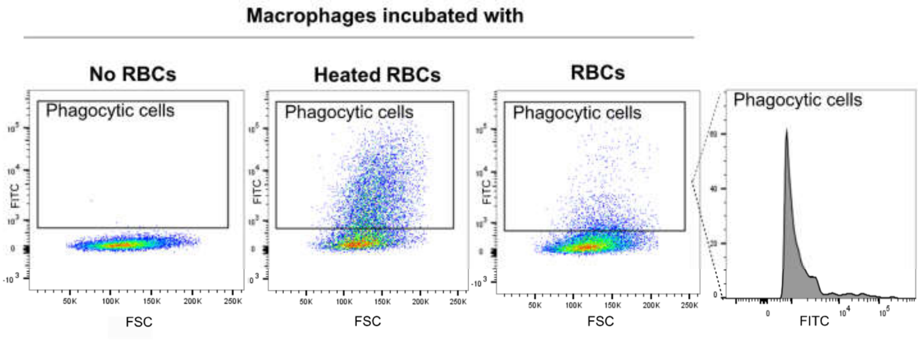

C.

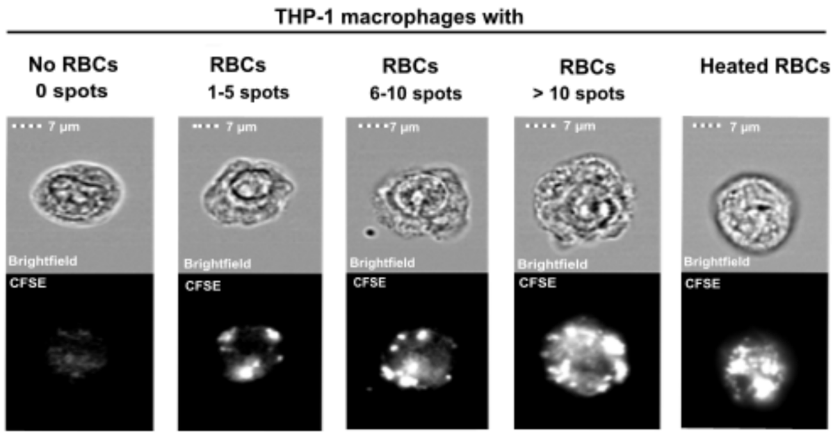

D.

	Number of spots (RBCs) in macrophages			
	0 spots	1-5 spots	6-10 spots	>10 spots
% of macrophages	75%	21%	2,58%	0,23%
GeoMFI	68 447	176 715	418 874	901 356
GeoMFI / Aera cell	26.37	62.44	138.16	260.67

Figure S1. The GeoMFI of CFSE+ macrophages related to the number of phagocytized RBCs. (A) To evaluate the erythrophagocytosis of GD RBCs by macrophages, RBCs were first stained with CFSE fluorescent dye. This panel shows a representative flow cytometry plot of CFSE-stained RBCs. **(B)** After 3h of co-incubation with macrophages, RBCs were removed and phagocytic cells were subjected to flow cytometry analysis. The percentage of CFSE+ cells was determined as well as the levels of fluorescence intensity within the CFSE+ population (average number of engulfed RBCs by macrophage). Heated RBCs were used as positive control. **(C)** Erythrophagocytosis events were observed in imaging flow cytometry using the ImageStream system (Amnis Corporation). Fluorescence have been quantified in individual cells in number of spots and represents CFSE stained RBCs that have been phagocytized by THP1-derived macrophages. The mask spot (M02-CFSE bright 16, 3, 1) 4 was used. **(D)** Repartition of macrophages showing 0 spot; 1-5 spots; 6-10 spots and >10 spots after representative erythrophagocytosis assay of RBCs. The GeoMFI associated to each sub-classes were quantified, as well as the GeoMFI normalized with the cell surface area.

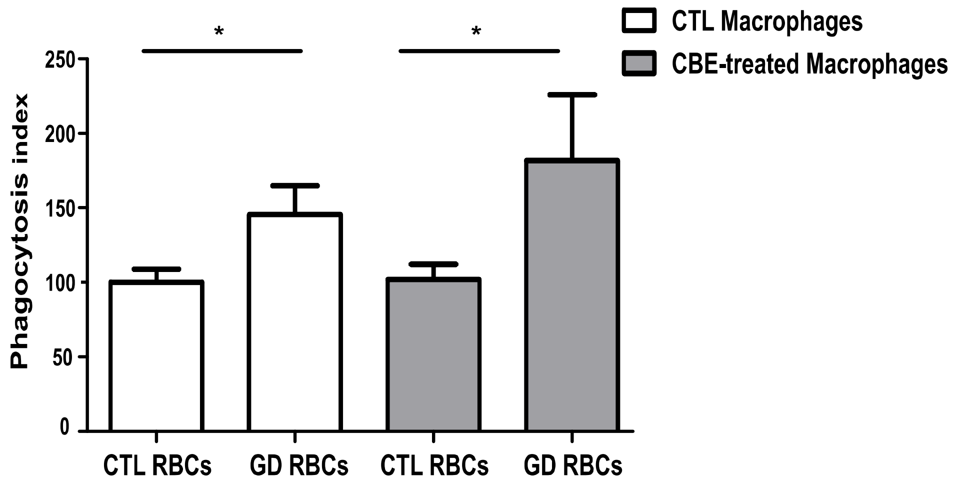

Figure S2. GD RBCs are more phagocytized by both CBE-treated and untreated macrophages than CTL RBCs. Erythrophagocytosis assays were performed using primary macrophages derived from healthy donor's monocytes. Macrophages were treated or not with CBE (1mM) during 6 days before the assay. They were then co-incubated with CTL RBCs or GD RBCs for 3 hours. The chart represents the phagocytosis index of CTL (n=4) and GD samples (n=4). Groups comparisons were performed using a t-test. *$p < 0.05$.

A.

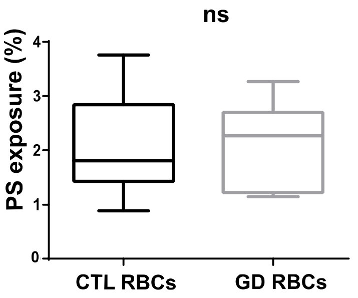

B.

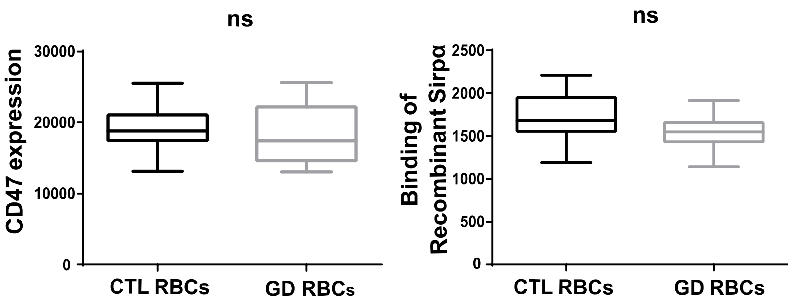

C.

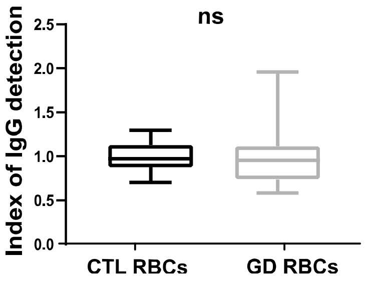

Figure S3. The uptake of GD RBCs is independent of phosphatidylserine (PS) exposure, CD47-Sirpα "don't eat me" signal and opsonizing immunoglobulins G. (A) The phosphatidylserine (PS) exposure was evaluated at the surface of the GD (n=12) and CTL (n=13) RBCs by flow cytometry. Group comparison was performed using an unpaired t-test. The medians are represented as horizontal bars; the upper and lower quartiles are represented as the top and the bottom of the box, respectively; and the maximum and minimum data values are shown by dashes at the top and the bottom, respectively, of the whiskers. ns : No statistically significant difference. **(B)** CD47 surface expression (left panel) and binding of recombinant Sirpα to CD47 surface (right panel) were evaluated by flow cytometry, using RBCs from GD patients (n=12) and healthy donors (n=13). The Y-axis represents the GeoMFI of cells in the PE and APC channels, respectively. Group comparison was performed using an unpaired t-test. The medians are represented as horizontal bars; the upper and lower quartiles are represented as the top and the bottom of the box, respectively; and the maximum and minimum data values are shown by dashes at the top and the bottom, respectively, of the whiskers. **(C)** Immunoglobulins G opsonization of RBCs was assessed by flow cytometry, using RBCs from GD patients (GD RBCs, n=18) and healthy donors (CTL RBCs, n=22). Group comparison was performed using an unpaired t-test. The medians are represented as horizontal bars; the upper and lower quartiles are represented as the top and the bottom of the box, respectively; and the maximum and minimum data values are shown by dashes at the top and the bottom, respectively, of the whiskers.

A.

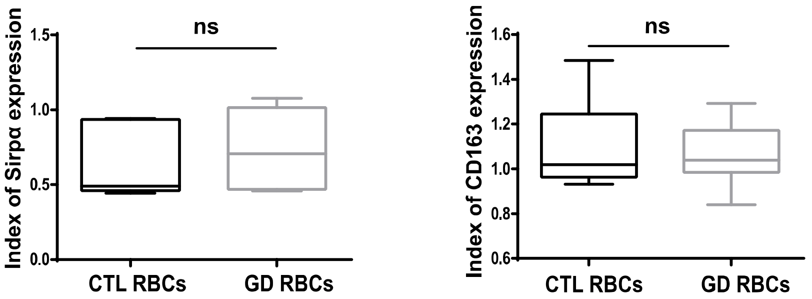

B.

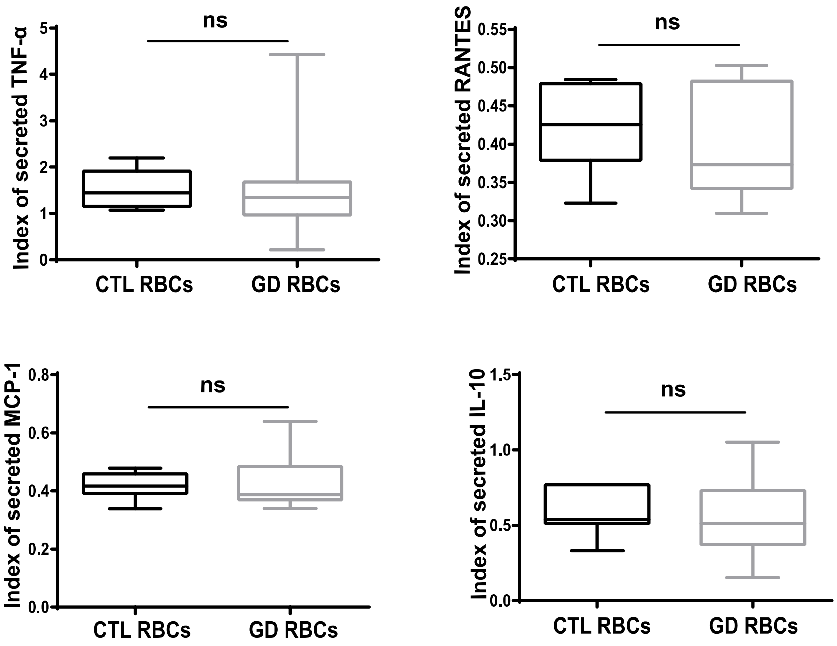

C.

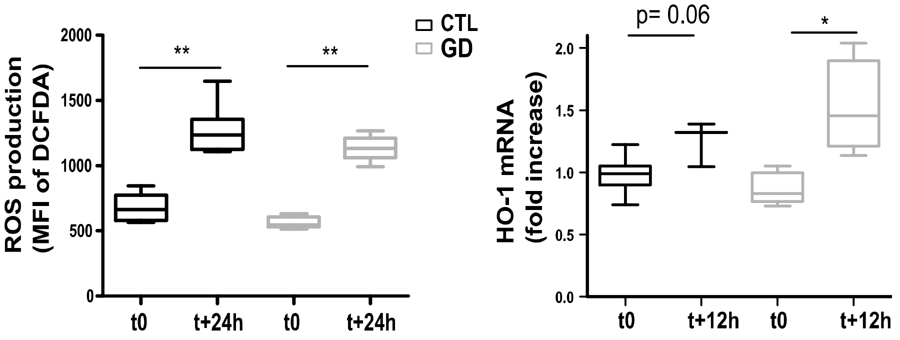

Figure S4. Phagocytosis of GD RBCs does not modify the expression of Sirp*α* and CD163 at the macrophage's cell surface, nor the secretion of TNF*α*, RANTES, MCP-1 pro-inflammatory cytokines, IL-10 anti-inflammatory cytokine, nor the ROS and Heme-oxygenase production. (A) The expression of different markers was investigated at the surface of THP1-derived macrophages after 12h of phagocytosis of CTL (n=11) or GD (n=13) RBCs. The expression levels of the phagocytic marker Sirp*α* and of the M2 polarization marker CD163 were evaluated by flow cytometry. The charts represent the index of marker expression. Group comparison was performed using a Mann-Whitney test. ns= non-significant. **(B)** Secretion levels of cytokines were measured by ELISA assay. The graphs represent an index of secreted cytokines in supernatant of CTL (n=12) or GD (n=13) RBCs engulfed macrophages.

The p values were determined by an unpaired t-test. ns= non-significant. **(C)** Left panel: Total ROS production was investigated in THP1-derived macrophages (n=6) after 3h hours of phagocytosis (t0) and 24h after phagocytosis (t+24h). The ROS production was detected by flow cytometry using H_2DCFDA. Enhanced ROS production is reflected by an increased GeoMFI in the FITC channel within the CFSE+ population. Group comparison was performed using a Kruskal-Wallis test. The medians are represented as horizontal bars; the upper and lower quartiles are represented as the top and the bottom of the box, respectively; and the maximum and minimum data values are shown by dashes at the top and the bottom, respectively, of the whiskers. $^{**}p < 0.01$. Right panel: Heme-oxygenase 1 mRNA quantification by qRT-PCR in macrophages before (t0) or 12h after phagocytosis (t+12h) of CTL (n=7) or GD (n=5) RBCs. Group comparison was performed two by two, using a Mann-Whitney test. The medians are represented as horizontal bars; the upper and lower quartiles are represented as the top and the bottom of the box, respectively; and the maximum and minimum data values are shown by dashes at the top and the bottom, respectively, of the whiskers. $^{*}p < 0.05$.

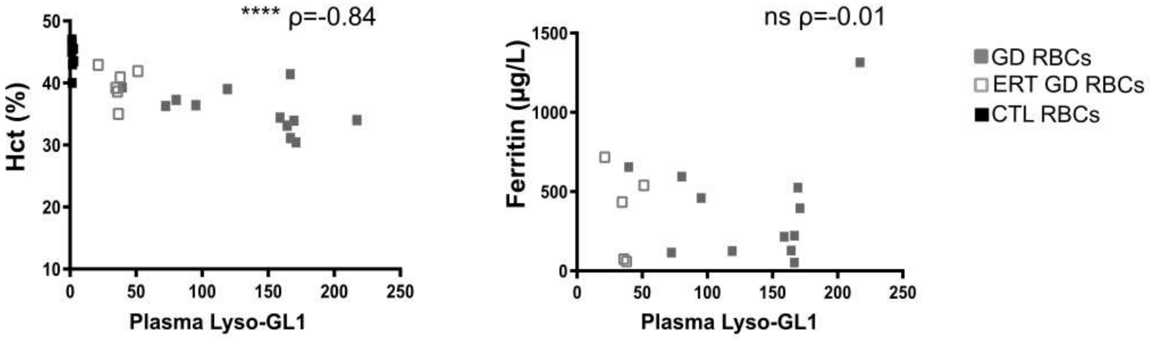

Figure S5. The plasma levels of Lyso-GL1 correlate with anemia but not correlate with ferritinemia. To assess the relationship between plasma Lyso-GL1 levels and hematological makers of GD activity, correlation analyses were carried out. The plasma Lyso-GL1 levels negatively correlated with the percentage of hematocrit (left panel), but did not correlate with levels of ferritin (right panel). GD, ERT GD and CTL RBCs values are depicted with grey, white and dark squares, respectively. The p and ϱ values were determined using the Spearman rank correlation test. ****$p < 0.0001$.

Table S1. Demographic characteristics, hematological and biological features between untreated (UT GD) and ERT-treated (ERT GD) patients.

	**UT GD**	**ERT GD**	***p* Value**
n	18	15	
Male	7	7	
Age (years)	19.5 [3-60]	20 [4-53]	
Mutations			
N370S heterozygous	13	11	
N370S homozygous	1	0	
D409H homozygous	1	1	
undeterminated	3	3	
Organomegaly			
**hepatomegaly**	11	1	0.0028
**splenomegaly**	16	2	< 0.0001
Bone involvement			
**disease history**	15	8	ns
**at sampling date**			
bone pain	2	2	ns
Radiologic skeletal manifestation	7	2	ns
Biological measurements			
**Hb** *(g/dL)*	12.4 [6.3-14]	14.4 [9.7-15.2]	0.0027
**Hct** *(%)*	36 [21-47]	40.9 [35-45]	0.0064
**Ferritin**	250 [53-1316]	72.5 [9-717]	ns
**Platelet count** *($10^3/mm^3$)*	94 [28-208]	184[37-250]	0.0009
**CCL18** *(pg/µmol)*	754 [149-3268]	146 [73-317]	0,0001
**Chitotrisidase activity** *(nmol/h/mL)*	13954 [517-19507]	727 [141-10240]	0,0001

The table recapitulates biological data from 18 untreated patients (UT GD) and 15 treated patients (ERT GD) for at least 6 months. We considered bone involvement as significant for patients with bone pains and/or radiologic (MRI) skeletal manifestations including bone infarcts, osteopenia, vertebral body collapses) at the time of sampling. The biological values are expressed as the median [extremes]. The *p* values were determined using the Mann-Whitney test or t-test to compare continuous variables between UT GD and ERT GD patients. Fisher's exact test was used to analyze categorical data in samples, i.e, splenomegaly and hepatomegaly. All characteristic and biological parameters, i.e, splenomegaly, hepatomegaly, Hb, Htc, ferritin, CCL18 (C-C motif chemokine ligand 18) and chitotriosidase activity were usually obtained on the day of blood sampling or within the previous few weeks. Analyses of RBCs properties, i.e, elongation index (EI) at 3Pa as well as the percentage of RBCs exhibiting abnormal morphologies were performed on fresh blood at the day of sampling. Hb: hemoglobin. Hct: hematocrit.

Table S2. Demographic characteristics, hematological and biological features of patients before their treatment (Pre-ERT) and after 6 to 42 months of ERT-treatment (Post-ERT).

n	10		
Male	3		
Age (years)	20 [3-52]		
Mutations			
N370S heterozygous	7		
N370S homozygous	0		
D409H homozygous	1		
undeterminated	2		
	**Pre-ERT**	**Post-ERT**	**p Value**
Organomegaly			
**hepatomegaly**	8	2	0,0055
**splenomegaly**	9	1	0,0001
Bone involvement			
**at sampling date**			
bone pain	2	2	ns
Radiologic skeletal manifestation	4	1	ns
Biological measurements			
**Hb** *(g/dL)*	12,55 [6,3-15,3]	14,25 [9,7-15,3]	0.0075
**Hct** *(%)*	36,35 [21-47]	40,05 [32-43,4]	0.0095
**Ferritin**	395 [64-655]	105 [9-717]	ns
**Platelet count** *(10^3/mm^3)*	69,5 [38-163]	159 [37-262]	0.0005
**CCL18** *(pg/µmol)*	765 [663-1110]	133 [73-317]	0.0156
**Chitotrisidase activity** *(nmol/h/mL)*	13582 [1470-21654]	435 [171-8765]	0.0039

The table recapitulates the characteristics and biological data for 10 GD patients before (Pre-ERT) and at least 6 months after post-ERT treatment (Post-ERT). We considered bone involvement as significant for patients with bone pains and/or radiologic (MRI) skeletal manifestations including bone infarcts, osteopenia, vertebral body collapses at the time of sampling. The biological values are expressed as the median [extremes]. The *p* values were determined using the Mann-Whitney test or t-test to compare continuous variables between UT GD and ERT GD patients. Fisher's exact test was used to analyze categorical data in samples, i.e, splenomegaly and hepatomegaly. All characteristic and biological parameters, i.e, splenomegaly, hepatomegaly, Hb, Htc, ferritin, platelet count, CCL18 (C-C motif chemokine ligand 18) levels and chitotriosidase activity were usually obtained on the day of blood sampling or within the previous few weeks. Hb: hemoglobin. Hct: hematocrit.

Methods S1

Macrophages differentiation. *THP1-derived macrophages.* The human monocytic THP1 cell line (ATCC® TIB-202™) was used to obtain macrophage-like cells (THP1-derived macrophages). THP1 cells were cultured in RPMI 10% fetal calf serum and differentiated into M0 macrophage-like cells with phorbol 12-myristate 13-acetate (PMA). THP1 cells were seeded in 12 well-plates (7.5 X 10^5 cells/well) and incubated for 48h at 37°C, 5% CO_2 in complete medium with 20ng/ml PMA. ***Blood-derived macrophages.*** $CD14^+$ circulating monocytes from whole blood of healthy donors were isolated using Whole Blood CD14 MicroBeads (Miltenyi Biotec). Sample purity was analyzed by flow cytometry using an APC- anti-CD14 antibody (clone M5E2, BD Biosciences) and the proportion of $CD14^+$ cells systematically reached over 95%. Isolated $CD14^+$ cells were seeded in 24 well-plates (7.5 X 10^5 cells/well) and differentiated into M2 polarized macrophages following incubation for 7 days at 37°C, 5% CO_2 in complete medium with 20ng/ml monocyte-colony stimulating factor (M-CSF, PeproTech). ***Gaucher patients blood-derived macrophages***. Peripheral blood mononuclear cells were isolated from whole blood from Gaucher patients or healthy donors and incubated at 37°C, 5% CO_2 in complete medium. The day after, adherent cells were differentiated into M2 polarized macrophages following incubation for 7 days at 37°C, 5% CO_2 in complete medium with 20 pg/ml for the first day, and then with 20 pg/ml of M-CSF. Macrophages were treated or not with CBE (1mM) during 6 days before the erythrophagocytosis assay.

Cellular phenotyping. The expression of surface markers on THP1 was assessed after 12h of erythrophagocytosis using the following antibodies (BD Biosciences); BV421-anti-CD1d (clone CD1d42), BB700-anti-HLA DP DQ DR (clone Tu39), APC-anti-CD36 (clone CB38) and PE-anti-CD163 (clone GHI/61). The expression of Sirpα was assessed using an APC- anti-CD172a antibody (clone 15-414, Molecular Probes). The surface expression of CD47 and the phosphatidylserine (PS) exposure on RBCs were assessed using an anti-CD47 antibody (clone 6H9C6, BD Biosciences) detected by a PE- anti-mouse antibody (Jackson ImmunoResearch), and FITC- lactadherin (Cryopep), respectively. After blocking with PBS 2% BSA for 30 minutes at 4°C, cells (7.5 X 10^5 THP-1 macrophages or 1 X 10^6 RBCs) were incubated with relevant antibodies or lactadherin in PBS 0.2% BSA for 30 min at 4°C in darkness. Samples data were acquired by flow cytometry using a FACSCanto II (BD Biosciences) and data were analysed using FlowJo software (Treestar). Internal controls (reference values) were used to normalize data between different experiments, resulting in an index of expression of the molecule.

Quantification of ROS production. ROS production in undifferentiated THP1 monocytes after erythrophagocytosis was measured using 2',7'-dichlorodihydrofluorescein diacetate (H_2DCFDA) (Molecular Probes), directly after erythrophagocytosis or 24h after removing non-phagocytized RBCs. Samples were then subjected to flow cytometry analysis.

Recombinant Sirpα binding to RBCs. One million RBCs were incubated during 30 min at RT in PBS 1% BSA with 50 µg/ml of recombinant biotinylated-Sirpα (BPS bioscience). Cell-bound biotinylated material was detected by flow cytometry using an APC-conjugated streptavidin (BD Biosciences).

Cytokines and chemokines quantification. Secretion of the human IL1-β, TNF-α, MCP-1, RANTES, IL-10 and CCL18 was measured in the macrophages' cell culture medium following erythrophagocytosis of CTL and GD RBCs. IL1-β, TNF-α, MCP-1, RANTES and IL-10 quantification was performed using Multiplex ProcartaPlex ELISA (ThermoFisher Scientific) according to manufacturer's instructions. Data was acquired on the Luminex MAGPIX® platform (xMAP® Technology). CCL18 quantification was performed using the human CCL18/PARC Quantikine ELISA kit (R&D Systems) according to manufacturer's instructions. Data was acquired on the iMark™ Microplate Absorbance Reader (Bio-Rad). Internal controls (reference values) were used to normalize data between different experiments, resulting in an index of secretion.

Reverse transcription and semi-quantitative real-time PCR. Total RNA was purified from thawed macrophages collected after phagocytosis using RNeasy Mini Kit (Qiagen, Courtaboeuf Cedex, France). RNA was reversed-transcribed by High Capacity cDNA Reverse Transcription Kit (Applied Biosystem – Thermofisher Scientific). Real-time PCR was carried out in a thermocycler (Biorad) by using iQ SYBR Green SuperMix (Biorad). Specific primers for qPCR were designed and evaluated for amplification efficiency. Forward and reverse primer sequences were respectively for HO-1, 5'- TTCAAGCAGCTCTACCGCTC -3'and 5'- GCAACTCCTCAAAGAGCTGGAT-3', for Hepcidin, 5'- GGCCAGCTGGATGCCCATGT-3' and 5'- TGACAGCAGCCGCAGCAGAA-3' and for the housekeeping genes Phosphoglycerate kinase 1 5'- TCTAACAAGCTGACGCTGGA -3', 5'-GACAGCAGCCTTAATCCTCTGGTT -3'. Normalization and quantification were calculated according to the formula $\mathbf{2^{\Delta\Delta Ct}}$ performed independently at least 5 times.
